# Environmental and economic impact of a vegan versus traditional mediterranean diet: OMNIVEG study

**DOI:** 10.1007/s00394-026-03939-3

**Published:** 2026-03-17

**Authors:** Miguel López-Moreno, Paula Marrero-Fernández, Carla Galiana, Millán Aguilar-Navarro, Alejandro Muñoz, Jorge Gutiérrez-Hellín, Ujué Fresán

**Affiliations:** 1 Diet, Planetary Health and Performance, Faculty of Health Sciences, Universidad Francisco de Vitoria, 28223 Madrid, Spain; 2 Institute of Health and Sport Sciences, Faculty of Health Science, Universidad Francisco de Vitoria, 28223 Madrid, Spain; 3 Exercise and Sport Science, Faculty of Health Sciences, Universidad Francisco de Vitoria, 28223 Pozuelo, Spain; 4 Institute of Agrifood Research and Technology (IRTA), Sustainability in Biosystems Research Program, Torre Marimon, 08140 Caldes de Montbui, Barcelona, Spain

**Keywords:** Mediterranean diet, Plant-based diet, Environmental impact, LCA, Food cost

## Abstract

**Background:**

Shifting dietary patterns toward more sustainable dietary practices is essential for addressing both chronic disease risk and environmental degradation. While the Mediterranean diet (MedDiet) is widely recognized for its health benefits, its environmental impact and cost may be higher than fully plant-based dietary patterns due to the inclusion of animal-derived foods. This secondary data analysis aimed to compare the environmental impacts and retail food costs of a traditional MedDiet and a vegan MedDiet, using dietary intake data from a controlled crossover trial.

**Methods:**

In the OMNIVEG study, 14 healthy, physically active men followed a traditional MedDiet for 3 weeks and a vegan MedDiet for 4 weeks, with a 1-week washout. Environmental impacts were assessed using Life Cycle Assessment while food costs were calculated from national retail price data.

**Results:**

The vegan MedDiet significantly reduced environmental impacts related to human health (− 54.5%), ecosystems (− 50.9%), and resource use (− 43.4%) compared to the traditional MedDiet (*p* < 0.01). Retail food cost was also reduced by 16.3% (*p* < 0.05). Differences were mainly attributable to the exclusion of animal-based foods; no significant differences in environmental impact were observed for shared food groups.

**Conclusions:**

Replacing animal products with plant-based foods in a Mediterranean dietary framework can enhance environmental sustainability and reduce food costs. These findings support the promotion of whole plant-based diets as a viable strategy for sustainable and affordable nutrition.

**Supplementary Information:**

The online version contains supplementary material available at 10.1007/s00394-026-03939-3.

## Introduction

Current dietary patterns in high-income countries are widely regarded as neither healthy nor environmentally sustainable, characterized by high intakes of animal products, added sugars, saturated fats, and salt, and low consumption of fruits, vegetables, legumes, and whole grains [1]. On the one hand, prevailing unhealthy diets are linked to approximately 11 million deaths globally each year [2]. In parallel, the current food system is a major contributor to environmental degradation, overexploitation of natural resources, and biodiversity loss [3]—factors that, in turn, negatively impact human health. Notably, it is estimated that around 23% of global deaths are attributable to modifiable environmental risk factors, including those related to externalities of the food system [4]. In this context, plant-based diets have gained increasing attention in recent years due to their potential to mitigate diet-related impacts on human health and environmental burden [5]. A substantial body of evidence supports the health benefits of whole plant-based diets, as well as their comparatively lower environmental impacts relative to current dietary patterns [6, 7]. Indeed, the widespread adoption of whole plant-based diets has been identified as essential for achieving a food system that remains within planetary boundaries [8, 9].

Plant-based diets encompass not only fully vegetarian diets but also those that include limited amounts of animal products, such as ovolactovegetarian, pescatarian, and even omnivorous diets low in animal products, like the traditional Mediterranean diet (MedDiet). Despite the overall benefits of plant-based diets compared to current dietary patterns, their effects can vary depending on the specific type of plant-based diet and the foods included within the pattern. A review pointed out that increasing the proportion of plant-based foods within plant-based diets may further enhance their health benefits [10]. On the other hand, although robust scientific evidence links lower consumption of animal products to reduced environmental impacts, previous studies have primarily focused on a limited set of environmental indicators—mainly greenhouse gas emissions, land use, and water use—thereby limiting their capacity to capture potential trade-offs across other relevant impact categories [11, 12]. Some frequently overlooked indicators, such as toxicity and the release of fine particulate matter are critical contributors not only to ecosystem degradation but also to the environmental burden on human health [13]. The few studies that consider a broader range of environmental indicators support the greater environmental sustainability and lower environmental human health damage of fully plant-based diets compared to dietary patterns that include limited amounts of animal products [14]. However, such studies typically analyze hypothetical, purpose-built dietary models rather than real-world consumption patterns, thereby limiting their external validity. Undoubtedly, a single, standardized model cannot adequately capture the diversity and flexibility inherent in plant-based dietary practices.

Equally important are the economic consequences of incentivizing further plant-based diets. The cost of a diet represents a critical and complex public health issue. Food prices play a significant role in shaping dietary choices, with the higher cost of certain healthier options often acting as a barrier to adopting more nutritious and sustainable eating patterns [15]. Animal-based foods, particularly meat and fish, generally exhibit higher food costs at retail compared to plant-based protein sources, such as legumes [16]. However, the higher cost of plant-based meat and dairy alternatives—which may facilitate the transition for individuals beginning to reduce their consumption of animal-based products [17]—could be a barrier for those attempting to adopt a more plant-based diet [18, 19]. In the same line, the environmental impacts of these products are higher than those of the raw ingredients that constitute them [20, 21]. Thus, their inclusion may attenuate the environmental benefits of further incentivizing more plant-based diets. Altogether, a comprehensive understanding of the health, environmental, and economic consequences associated with encouraging greater consumption of plant-based diets is crucial for developing effective public health policies that support sustainable and equitable dietary transitions.

Recently, the OMNIVEG study found that transitioning from a traditional MedDiet to a vegan MedDiet—a plant-exclusive adaptation of the traditional MedDiet that preserves the core principles by emphasizing whole plant-based foods and healthy fats, mainly from virgin olive oil and nuts—in healthy, physically active men in Spain led to reductions in total cholesterol, low-density lipoprotein cholesterol, blood pressure, and the neutrophil-to-lymphocyte ratio [22]. To shed light on the environmental and economic consequences of further promoting a more plant-based MedDiet in Spain, this secondary analysis of the OMNIVEG trial aimed to compare the environmental impacts and retail food costs of vegan and traditional MedDiets.

## Materials and methods

### The OMNIVEG study

The methodology of the OMNIVEG study has been previously detailed [22]. In brief, this study was a controlled crossover trial conducted between June and August 2023 in Madrid, Spain. Physically active males initially adhered to a traditional MedDiet for three weeks, then switched to a vegan MedDiet for four weeks, with a 7-day washout period between the two phases. The study protocol and design were approved by the Ethics Committee of the University Francisco de Vitoria (20/2023), and it fully complied with the 1964 Helsinki Declaration and its subsequent modifications (last update 2013). The trial was registered on ClinicalTrials.gov (ID: NCT06008886).

### Participants

The sample size was calculated using G*Power^®^ software (3.1.9.7; Heinrich Heine University, Düsseldorf, Germany). As this analysis represents a secondary outcome, the sample size calculation was based on the primary outcome reported in a previous study [22], considering low-density lipoprotein cholesterol and effect sizes reported in prior research evaluating the impact of a vegan diet on this variable [23, 24]. Considering the large effect sizes reported in previous studies, a medium effect size of d = 0.5 was used for the sample size calculation. For a difference between two dependent means design, a statistical power of 80% and an α error probability of 0.05, at least 14 participants were needed. Considering a potential dropout rate of 10%, the final sample size was set at 17 participants. Recruitment was done via flyers on the Universidad Francisco de Vitoria campus (Madrid, Spain) and social media advertising. Inclusion criteria included men aged 18–40, physically active (moderate-intensity exercise 3–5 days/week as per WHO recommendations), with a BMI of 18.5–24.9 kg/m^2^, no tobacco use, minimal alcohol consumption, and no orthopedic limitations. Participants with chronic cardiovascular, metabolic, gastrointestinal, respiratory, or musculoskeletal diseases, as well as those with serious musculoskeletal injuries in the last six months, were excluded. Screening was conducted using a pre-participation questionnaire to collect medical and exercise histories. Before the enrollment, participants were informed about the potential risks and discomforts associated with the study and provided written informed consent to participate voluntarily.

### Dietary intervention

Prior to the dietary intervention, participants underwent an initial dietary evaluation in the laboratory conducted by a registered dietitian to evaluate their body characteristics and dietary patterns. Based on this assessment, a normocaloric traditional MedDiet was designed for a 3-week intervention. Participants were already following a Mediterranean-style diet at baseline, and this period aimed to standardize energy intake and macronutrient distribution. The diet emphasized a high intake of whole plant-based foods, moderate to low consumption of fish, poultry, low-fat dairy products, and eggs, minimal red and processed meat intake, and exclusion of sweets. Olive oil and nuts was the main added fat, and animal protein accounted for 60% of total protein intake [7]. Afterwards, participants followed a 7-day washout period. No specific dietary instructions were provided for this period and participants were asked to resume their habitual diet prior to the standardized intervention. Subsequently, participants transitioned to a vegan version of the MedDiet for four weeks. The vegan MedDiet excluded dairy products, eggs, fish/seafood, and meat, which were substituted with plant-based protein sources characteristic of the Mediterranean pattern, such as legumes and nuts. While the vegan diet consisted predominantly of whole plant-based foods, any use of nutritionally equivalent plant-based meat and dairy alternatives occurred only to a minimal extent to facilitate a smoother transition toward a vegan MedDiet (Table S1). To address the potential vitamin B12 deficiency resulting from the vegan diet, participants received 1000 µg of cyanocobalamin (vitamin B12) twice a week (Harrison Sports Nutrition, Granada, Spain) [25].

Dietary intake during the intervention was monitored through three weekly 24-hour dietary recalls (two on non-consecutive weekdays and one on a weekend day), in which participants recorded the foods consumed along with their weights and/or household measurements. Energy and nutrient intakes were quantified from food consumption data using specialized analytical software (Dietopro, Valencia, Spain) [26]. Participants received continuous feedback from the researchers to address any questions and ensure proper adherence. Participants who did not adhere to the prescribed range of servings for each food group representative of the respective dietary intervention for more than one day were excluded from the analysis (Table S1) (*n* = 1 for the traditional MedDiet and *n* = 1 for the vegan MedDiet).

### Environmental impact assessment

The dietary environmental impact was assessed using Life Cycle Assessment (LCA) methodology, following the ISO 14040/44:2006 standards [27, 28]. LCA is a systematic method for quantifying the environmental impacts associated with all stages of a product’s life cycle, including materials, processes, organizations, or human activities.

The functional unit was defined as the daily diet in mass-based units, encompassing all recorded foods. System boundaries were established from cradle to fork, including pre- and on-farm activities, processing, packaging, transportation, storage, as well as cooking. Food loss and waste within the system boundaries, as well as packaging waste management, were accounted for.

Agribalyse v3.1.1 served as the primary Life Cycle Inventory (LCI) database (see [29] for details). Each food item reported in the food diaries was matched to its corresponding LCI entry in the Agribalyse database. Adjustments were made to tailor the LCI data to the specific needs of this study, particularly regarding recipes and cooking methods. Some food items were reported in non-mass units, such as volume or standard household serving sizes. To enable conversion, the following densities were assumed: 0.92 g/ml for oil, and 1.034 g/ml for milk and plant-based drinks. Standardized beverage volumes included 250 ml for a glass of water, 30 ml for a cup of coffee, and 100 ml for a cup of infusion. In a few cases, uncooked weights were reported for cereals and legumes; to account for cooking-related weight changes, raw-to-cooked conversion factors of 2.259 and 2.33 were applied, respectively.

LCI characterization was conducted using the ReCiPe 2016 v1.1 method, employing a hierarchist perspective [30]. ReCiPe 2016 was selected due to its widespread acceptance and application within the LCA community. This study primarily focused on the damage of three areas of protection (endpoint indicators), namely human health, ecosystems, and resource scarcity. Focusing on endpoint indicators facilitate the interpretation of the findings. Nevertheless, the 18 midpoint environmental indicators (i.e., global warming, stratospheric ozone depletion, ionizing radiation, ozone formation-human health, fine particulate matter formation, ozone formation-terrestrial ecosystems, terrestrial acidification, freshwater eutrophication, marine eutrophication, terrestrial ecotoxicity, freshwater ecotoxicity, marine ecotoxicity, human carcinogenic toxicity, human non-carcinogenic toxicity, land use, mineral resource scarcity, fossil resource scarcity, and water consumption), on which those areas of protection are based, were also individually assessed. The IMPACT World+ v2.1 method was applied as a sensitivity analysis for the endpoint indicators [31, 32]. Conducting this sensitivity analysis was essential to assess the influence of selecting a specific Life Cycle Impact Assessment (LCIA) method. We specifically selected IMPACT World+ v2.1 because it is one of the most updated LCIA methods available, and due to its focus on endpoint (damage) indicators. The sensitivity analysis was specifically limited to the human health and ecosystem endpoints, given that IMPACT World+ does not include characterization factors for resource scarcity.

### Cost at retail assessment

For the assessment of food costs, the methodology employed in previous studies was applied [33, 34]. Dietary intake data from the records were linked to a retail food cost database provided by the Ministry of Agriculture, Fisheries, and Food [35]. Since the Ministry**’**s database provides costs for foods as purchased (including inedible parts), and the dietary intake data reflect the edible portion of foods as consumed, the retail costs were adjusted to the edible fraction and raw-to-cooked ratio by applying appropriate conversion factors [36]. Retail prices used in the analysis correspond to data representative of Madrid, the region where the study was conducted. The total daily food costs were calculated by multiplying the cost per kilogram (€ per kilogram) of each food item during the intervention months by the daily quantity consumed through dietary recalls. When a dietary recall item comprised multiple ingredients, the total cost was estimated using the weighted average of the ingredients’ prices based on their amounts.

### Statistical analysis

Continuous variables, representing daily values, were reported as means with standard deviations (SD) for the entire sample. The normality of each variable’s distribution was assessed using the Shapiro-Wilk test, and parametric tests were applied as all variables were normally distributed. A paired-sample Student’s t-test was conducted to assess differences in the average dietary intake, environmental impact and daily food costs between groups. The effect size was assessed with Cohen’s d, and the following criteria were established: trivial (0-0.19), small (0.20–0.49), medium (0.50–0.79) and large (≥ 0.80) [37]. The significance level was set at *p* < 0.05. The statistical analysis was performed using IBM Statistical Package for Social Sciences (SPSS) version 22.0 (IBM, Chicago, IL, USA) and Prism 10.0 software (GraphPad Software, San Diego, CA, United States).

## Results

### Participants

**Fig. 1:**
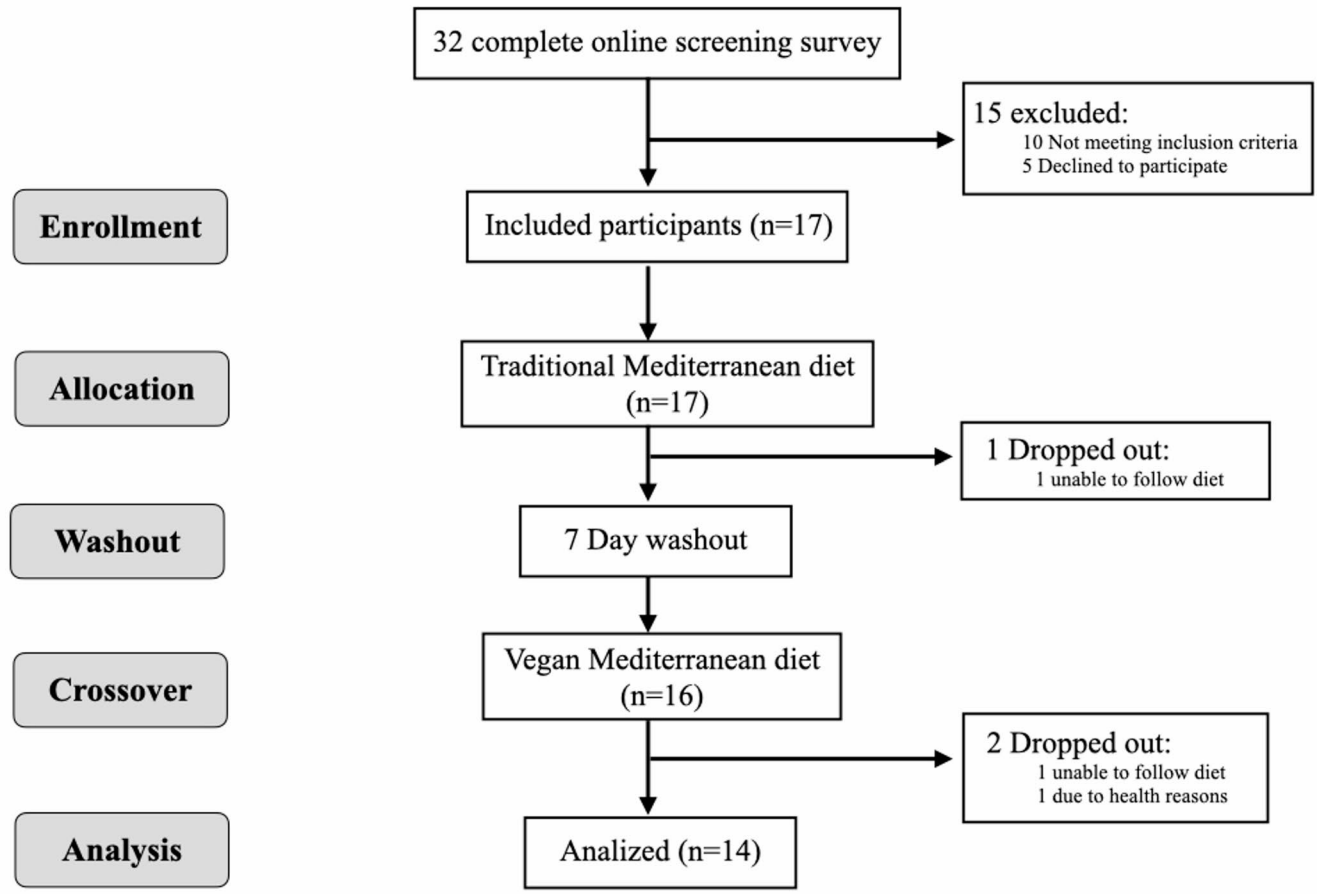
Flow diagram of participants through the study

Thirty-two individuals were initially screened for eligibility. Of these, ten did not meet the inclusion criteria, and five declined to participate following the initial interview. Seventeen participants began the intervention protocols. One participant withdrew during the traditional MedDiet phase, while two participants dropped out during the vegan MedDiet phase. Finally, the study sample consisted of 14 participants (mean age 24.6 ± 7.0 years, range 18–37) (Fig. 1).

### Dietary assessments

During the vegan phase, participants consumed significantly higher amounts of legumes, grains, nuts, seeds, plant-based dairy alternatives, plant-based meat alternatives, and potatoes (*p* < 0.05). No significant differences were observed in the consumption of fruits, vegetables, olive oil, or sweets between the dietary interventions (*p* > 0.05) (Table S2). At the nutritional level, the only difference observed was in fibre intake, which was higher in the vegan version (41.1 ± 4.9 g/day) compared to the traditional MedDiet (30.7 ± 2.8 g/day), and in total daily protein intake and percentage of energy from protein (*p* < 0.01) (Table S2). However, body weight–adjusted protein intake did not differ significantly between the traditional Mediterranean diet (1.6 g/kg/day) and the vegan Mediterranean diet (1.6 g/kg/day) (*p* = 0.239).

### Environmental and economic impacts of dietary interventions

**Fig. 2:**
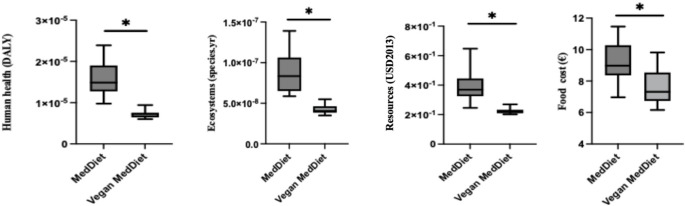
Environmental impact and food cost reductions of a vegan vs. traditional MedDiet

The vegan MedDiet led to significantly lower impacts on environmental human health burden (54.5%; 95% confidence interval (CI): 49.4–59.6%), ecosystems (50.9%; 95% CI: 45.5–56.5%), resources (43.4%; 95% CI: 38.2–48.7%), and food cost (16.3%; 95% CI: 1.4–31.2%) compared to the traditional MedDiet (*p* < 0.05) (Fig. 2). In the sensitivity analysis, significant differences between diets were also observed for human health reduced by 44.3% (95% CI: 39,7.5–49.6%), and ecosystem reduced by 37.2% (95% CI: 31.2–41.2%) endpoint indicators (*p* < 0.01). The vegan MedDiet resulted in a significant reduction across all mid-point environmental impact outcomes analyzed (*p* < 0.01), with reductions ranging from 14.6% to 70.7%, for terrestrial ecotoxicity and terrestrial acidification, respectively (Table 1).

**Table 1:** Mid-point environmental impacts of dietary interventions, assessed via Life Cycle Assessment using ReCiPe.2016 v1.1 characterization method

	MedDiet	Vegan MedDiet	*p*-value
*Mid-point*			
Global warming (kg CO_2_ eq)	6.26	2.57	< 0.001
Stratospheric ozone depletion (kg CFC11 eq)	3.60 × 10^−5^	1.36 × 10^−5^	< 0.001
Ionizing radition (kBq Co-60-eq)	1.58	8.26 × 10^−1^	< 0.001
Ozone formation, Human health (kg NOx eq)	1.77 × 10^−2^	6.85 × 10^−3^	< 0.001
Fine particule matter formation (kg PM 2.5 eq)	1.07 × 10^−2^	3.76 × 10^−3^	< 0.001
Ozone formation, Terrestrial ecosystem (kg NOx eq)	1.81 × 10^−2^	7.02 × 10^−3^	< 0.001
Terrestrial acidification (kg SO_2_ eq)	4.9 × 10^−2^	1.44 × 10^−2^	< 0.001
Freshwater eutrophication (kg P eq)	1.9 × 10^−3^	1.18 × 10^−3^	< 0.001
Marine eutrophication (kg N eq)	1.07 × 10^−2^	3.84 × 10^−3^	< 0.001
Terrestrial ecotoxicity (kg 1,4-DCB)	27.61	23.6	0.001
Freshwater ecotoxicity (kg 1,4-DCB)	2.33 × 10^−1^	1.68 × 10^−1^	< 0.001
Marine ecotoxicity (kg 1,4-DCB)	2.86 × 10^−1^	2.00 × 10^−1^	< 0.001
Human carcinogenic toxicity (kg 1,4-DCB)	2.31 × 10^−1^	1.29 × 10^−1^	< 0.001
Human non-carcinogenic toxicity (kg 1,4-DCB)	9.14	7.55	< 0.001
Land use (m^2^a crop eq)	5.82	3.14	< 0.001
Mineral resource scarcity (kg Cu eq)	3.56 × 10^−2^	2.40 × 10^−2^	< 0.001
Fossil resource scarcity (kg oil eq)	1.40	6.11 × 10^−1^	< 0.001
Water consumption (m^3^)	3.41 × 10^−1^	2.84 × 10^−1^	< 0.001

The data are shown as mean daily values. Eq, equivalents; MedDiet, Mediterranean diet; Vegan MedDiet, Vegan Mediterranean diet. Comparison between groups by paired Student’s t-test

End-point environmental and economic impacts associated with each food group are presented in Table S3. Differences between the traditional MedDiet and the vegan MedDiet were observed only in the food groups that characterize each diet (i.e., those predominantly consumed in one of the interventions), namely meat, fish, eggs, dairy products, plant-based meat and dairy alternatives and legumes (*p* < 0.05). In contrast, no differences were found for food groups equally representative in both diets, such as fruits, and vegetables. Despite a higher intake of cereals and nuts, their consumption within the vegan MedDiet did not lead to increased environmental impacts compared to that within the traditional MedDiet; notably, the increased cereal consumption did not incur additional costs (*p* < 0.01).

## Discussion

This study compared the environmental and economic impacts of a traditional MedDiet and a vegan MedDiet in a group of healthy, physically active men. The results show that the vegan MedDiet significantly reduced all environmental impacts assessed, as well as overall food cost, compared to a traditional MedDiet. These reductions were largely attributable to the exclusion of animal-based products—such as meat, fish, eggs, and dairy—and their replacement with plant-based options rich in fiber. These findings complement previous publications from the OMNIVEG project [22, 38], which observed improvements in cardiometabolic markers with the vegan MedDiet.

Our findings align with previous research showing that reducing the intake of animal products is associated with a lower overall dietary environmental impact. This association has been consistently observed in both modeling and observational studies and applies not only to Spanish dietary patterns but also to those in other regions [9, 11, 39–41]. In our study, although the caloric contributions of animal products within the Mediterranean diet were relatively low (18.5%), their replacement with plant-based foods led to disproportionately large reductions—between 40% and 50%—in composite environmental impact indicators. These values are consistent with previous studies comparing Mediterranean and vegan diets [40]. In addition, although plant-based meat alternatives were not explicitly prescribed in the intervention, they were consumed in small amounts within the vegan Mediterranean diet group. While the environmental impact of these products is higher than that of their whole-food plant-based versions, it remains substantially lower than that of animal-based meat, reinforcing the overall environmental advantages observed in the vegan Mediterranean pattern [21, 42]. This suggests that even moderate consumption of animal-based foods can carry a substantial environmental burden. Or in the other way around, that modest reductions in the intake of certain animal products may yield significant environmental benefits.

Beyond the composite indicators, our study also found reductions across all individual environmental indicators analyzed when following the vegan MedDiet. This is particularly relevant given that trade-offs between environmental outcomes have been reported in previous research [12, 43–45]. While there is broad consensus that diets excluding animal products are generally more environmentally sustainable, the effect of vegan diets on blue water use remains a topic of debate. Modeling studies often estimate that the complete exclusion of animal products increases blue water consumption [9, 40]. In contrast, several studies—including the present and those targeting the EPIC-Oxford cohort, one of the largest with a high proportion of vegans [46]—have reported reductions in dietary water use, alongside improvements in other environmental indicators. These discrepancies between modeling and observational evidence may be attributed to the specific food substitutions assumed in modeling scenarios. For example, a global modeling study replaced animal products not only with plant-based protein sources but also with substantial amounts of fruits and vegetables, which are typically high water-intensive [47]. On the other hand, when both green and blue water footprint are considered, animal-source foods stand out among the most water-intensive food products [48]. This point is increasingly relevant, as the planetary boundary for green water use has already been exceeded, underscoring the importance of incorporating green water considerations into environmental assessments [49]. In any case, although vegan diets may offer the potential for the lowest dietary blue water footprint, it is important to emphasize that mitigating the food system’s contribution to freshwater scarcity will primarily rely on advancements in agricultural practices and water management, rather than on dietary changes [9]. As such, concerns about potential increases in blue water use should not be considered a barrier to the promotion or adoption of more plant-based diets—particularly given the substantial evidence supporting their benefits across other key environmental domains.

Scientific literature consistently identifies meat and dairy products—and to a lesser extent, fish—as major contributors to the environmental impact of diets [14, 50, 51]. In our study, meat and fish were key contributors, whereas dairy products were not. This is likely due to moderate consumption levels and a preference for lower-impact options such as milk and yogurt over hard cheese [45]. The identification of fish as a significant contributor is particularly relevant given its central role in the Mediterranean diet. Although the environmental impact of fish varies widely by species, previous studies have shown that some have environmental footprints comparable to those of meat products [14, 45, 52]. Despite this, fish has not received the same level of scrutiny in scientific discussions on sustainable diets—likely due to its reputation as a healthy source of protein. In fact, it is often promoted, rather than limited, in sustainable diets as a substitute for meat [52]. However, current evidence does not indicate that fish offers greater health benefits than plant-based protein sources [53]. On the contrary, studies suggest that a fully plant-based Mediterranean diet may lead to greater health benefits than its traditional omnivorous counterpart [10]. In line with this, modeling studies indicate that achieving a more nutritionally balanced, healthy and environmentally sustainable diet in Spain would require at least a partial substitution of both meat and fish with plant-based alternatives [51]. Altogether, the promotion of fish within the omnivorous Mediterranean diet should be reconsidered in the pursuit of truly sustainable and health-promoting dietary patterns.

Regarding retail food costs, the vegan MedDiet was associated with an approximate 16% reduction in total food expenses compared to the traditional MedDiet. This finding aligns with previous studies reporting that vegetarian dietary patterns are generally less expensive than other reference diets, such as the MedDiet and Dietary Approaches to Stop Hypertension [41, 54, 55]. This cost reduction is largely driven by the substitution of animal protein sources. Modelling studies have also shown that replacing animal protein foods with legumes can result in significant cost savings [56]. Unlike previous studies, the diets analyzed in the present study also included some plant-based meat and dairy analogs. Despite the higher cost of these alternatives, the potential price increment was fully offset by the savings from using legumes as the primary protein source instead of animal-derived. Additionally, although participants spent more on nuts in the vegan version, these over costs were largely balanced by the removal of high-cost items such as fish and meat, resulting in no overall increase (even decrease) in total diet cost. The observed reduced retail food cost associated with the vegan MedDiet contrasts with common consumer perceptions that whole plant-based diets are inherently more expensive [57]. Enhancing public awareness of the potential economic benefits of plant-based diets may support broader acceptance and leverage the transition to more sustainable eating patterns [58].

The present study is strengthened by the use of actual dietary intake data collected under controlled intervention conditions, with comparable total energy and macronutrient intake between the two dietary patterns. This enhances the ecological validity of the findings compared to modelling studies based on hypothetical scenarios or population-level estimates. Moreover, the diets were tailored to participants’ individual preferences and nutritional requirements, which may facilitate long-term adherence beyond the research setting. Another important strength is the application of the LCA methodology, incorporating both mid-point and end-point indicators. This comprehensive approach allows for a robust evaluation of environmental outcomes across multiple domains, including areas of protection such as human health, ecosystems, and resources. However, several limitations should be acknowledged. First, as this analysis represents a secondary outcome, the sample size calculation was based on the primary outcome reported in a previous study. Although this could theoretically increase the risk of a type II error for the secondary outcomes, differences were detected in the variables included in this analysis, suggesting that this limitation did not materially affect the findings. Second, the sample was limited to young, healthy, physically active men, which may restrict generalizability to other populations. In this line, the relatively higher protein and carbohydrate intakes compared to those prescribed within the Mediterranean diet to the general population reflect the physically active nature of the study population, in line with current sports nutrition recommendations [59]. Besides, the overall food group distribution remained consistent with commonly accepted definitions of the Mediterranean diet. Additionally, the macronutrient distribution was matched across both dietary patterns, ensuring that between-diet comparisons were not confounded by differences in protein or carbohydrate intake. We acknowledged, however, that given that protein-rich foods—particularly of animal origin—are among the main contributors to environmental impact in omnivorous diets, higher protein intakes may have amplified differences in environmental indicators compared with diets containing lower protein amounts. However, this does not necessarily limit the generalizability of the findings, particularly in light of the consistent evidence reported across diverse populations in previous research. Third, plant-based dairy and meat alternatives are not traditionally part of the Mediterranean dietary pattern. However, they were included to support dietary adherence, as all participants were omnivorous at baseline. Nevertheless, all of them were minimally processed or fortified options (e.g., calcium-fortified soy or oat drinks, soy-based yogurts, and legume-based meat alternatives), and their overall contribution to total energy and protein intake was low (on average, 10.4% of total protein intake in the vegan Mediterranean diet). Forth, although vitamin B12 supplementation was provided in the vegan diet group, this reflects current clinical recommendations for vegan diets rather than masking a deficiency [60]. Fifth, in the absence of a national food environmental impact database, the main LCI database used in this study was developed to reflect the French food system. Nevertheless, we partially adapted LCIs to the requirements of this study by modifying inventories when necessary. Sixth, food cost estimates were based on regional retail prices during the intervention period and may not reflect broader economic factors, including seasonal variability, within-region price differences, or variations between retail outlets and local markets. Moreover, other consumers’ costs—such as transportation from retail to home, food storage, and meal preparation—were not considered, which may affect the real-world applicability of the cost estimates. Future research should investigate the long-term acceptability, cost-effectiveness and environmental impact of promoting more plant-based dietary patterns in our context, particularly in more diverse populations and under varied socioeconomic and geographic conditions.

## Conclusion

This analysis of the OMNIVEG trial suggested that adopting a vegan MedDiet resulted in substantial reductions in environmental impacts as well as a decrease in food cost, compared to a traditional MedDiet. These differences were primarily driven by the exclusion of animal-based foods, with no significant changes observed in food groups common to both diets. These findings highlight the importance of increasing the proportion of plant-based foods as a transition to a healthy and sustainable dietary pattern, while also being a cost-effective dietary approach. Besides the potential benefits, further long-term studies are warranted to confirm these findings and evaluate adherence to such dietary patterns, while also considering the potential implications for nutritional status when shifting to a vegan MedDiet.

## Supplementary Information

Below is the link to the electronic supplementary material.

Supplementary Material 1

Supplementary Material 2

Supplementary Material 3
