# Supplementary material for: Environmental and economic impact of a vegan versus traditional mediterranean diet: OMNIVEG study
Source: Eur J Nutr. 2026 Mar 17;65(3):97. doi: 10.1007/s00394-026-03939-3 (PMC12995930; doi:10.1007/s00394-026-03939-3)
Supplement: Supplementary file 1 — Supplementary Material 1 [file 394_2026_3939_MOESM1_ESM.docx]

Table S1. Composition Traditional Mediterranean diet and vegan Mediterranean diet

| Servings | **Traditional Mediterranean diet** | **Vegan Mediterranean diet** |
| --- | --- | --- |
| Fruit | 1-2 servings/main meal | 1-2 servings/main meal |
| Vegetables | ≥ 2 servings/main meal | ≥ 2 servings/main meal |
| Cereals^a^ | 1-2 servings/main meal | 1-3 servings/main meal |
| Olive oil | 1servings/main meal | 1servings/main meal |
| Olives/Nuts/Seeds | 1-2 servings/day | ≥ 2 servings/day |
| Dairy products^b^ | 2 servings/day | - |
| Plant based dairy alternatives | - | 2 servings/day |
| Legumes | ≥ 2 servings/week | ≥ 5 servings/week |
| Eggs | 2-4 servings/week | - |
| Fish/seafood | ≥ 2 servings/week | - |
| White meat^c^  Red meat^d^  Processed meat  Plant based meat alternatives  Potatoes  Sweets^e^ | 2 servings/week  < 2 servings/week  ≤ 1 servings/week  -  ≤ 3 servings/week  ≤ 2 servings/week | -  -  -  ≤ 3 servings/week  ≤ 2 servings/week |

*Main meals: breakfast, lunch and dinner. ^a^ Bread, pasta, couscous and other cereals (preferably whole grain) ^b^ Milk, yoghurt, cheese, ice-cream (preferably low fat) ^c^ Chicken, turkey or rabbit

^d^ Pork, beef, veal, lamb, mutton or goat ^e^ Sugar, candies, pastries, sweetened fruit juices and soft drinks
