# Supplementary material for: Environmental and economic impact of a vegan versus traditional mediterranean diet: OMNIVEG study
Source: Eur J Nutr. 2026 Mar 17;65(3):97. doi: 10.1007/s00394-026-03939-3 (PMC12995930; doi:10.1007/s00394-026-03939-3)
Supplement: Supplementary file 2 — Supplementary Material 2 [file 394_2026_3939_MOESM2_ESM.docx]

**Table S2**. Dietary characteristics of the traditional and vegan Mediterranean diets.

|  | **Traditional Mediterranean diet** | **Vegan Mediterranean diet** | **P-value** |
| --- | --- | --- | --- |
| ***Servings/day*** |  |  |  |
| Fruit | 1.4 (0.4) | 1.6 (0.6) | 0.31 |
| Vegetables | 2.3 (0.6) | 2.4 (0.3) | 0.58 |
| Cereals^a^ | 1.4 (0.4) | 2.9 (0.1)* | **<0.01** |
| Olive oil | 1.1 (0.1) | 1.0 (0.2) | 0.11 |
| Olives/Nuts/Seeds | 1.1 (0.3) | 2.3 (0.3)* | **<0.01** |
| Dairy products^b^ | 2.5 (1.1)* | - | **<0.01** |
| PBDA | 0.08 (0.0) | 2.0 (0.8)* | **<0.01** |
| ***Servings/week*** |  |  |  |
| Legumes | 1.7 (0.3) | 7.6 (1.8)* | **<0.01** |
| Eggs | 3.0 (1.0)* | - | **<0.01** |
| Fish/seafood | 1.5 (0.8)* | - | **<0.01** |
| White meat^c^ | 2.5 (1.1)* | - | **<0.01** |
| Red meat^d^ | 1.1 (0.2)* | - | **<0.01** |
| Processed meat | 1.3 (1.1) | - | **<0.01** |
| PBMA | - | 2.3 (1.5)* | **<0.01** |
| Potatoes | 2.0 (0.4) | 3.2 (0.5) | **<0.01** |
| Sweets^e^ | 1.3 (0.3) | 1.5 (0.5) | 0.20 |
| Nutrients intake |  |  |  |
| Energy, kcal/day | 2599.6 (180.8) | 2634.9 (148.3) | 0.14 |
| CHO, g/day | 311.3 (29.7) | 320.8 (52.4) | 0.39 |
| CHO, % of energy | 47.9 (3.3) | 48.9 (5.1) | 0.17 |
| Sugar, % of energy | 13.3 (1.1) | 14.2 (0.8) | 0.21 |
| Dietary fibre, g/day | 30.7 (2.8) | 41.1 (4.9) | **<0.01** |
| Protein, g/kg/day | 1.6 (0.1) | 1.6 (0.1) | 0.239 |
| Protein, g/day | 120.2 (5.1) | 115.3 (4.8) | **<0.01** |
| Protein, % of energy | 18.5 (1.5) | 17.5 (0.9) | **<0.01** |
| Fat, g/day | 97.0 (17.8) | 99.0 (13.2) | 0.62 |
| Fat, % of energy  Saturated fat, % total fat energy | 33.6 (5.2)  25.2 (6.8) | 33.8 (2.1)  13.6 (4.4) | 0.90  **<0.01** |
| Monounsaturated fat, % total fat energy | 59.1 (7.2) | 58.0 (5.9) | 0.49 |
| Polyounsaturated fat, % total fat energy | 15.7 (4.0) | 28.4 (6.1) | **<0.01** |

All values are means (standard deviation). Values reflect dietary intake based on three 24-hour recalls per week (two weekdays, one weekend day). Statistical comparison between diets was performed using paired Student’s t-test. Asterisks (*) indicate statistically significant differences between diets (p < 0.05).

*a* Includes bread, pasta, couscous, cereals (preferably whole grain), and pseudocereals (e.g., quinoa, amaranth, buckwheat).
*b* Milk, yoghurt and cheese.
*c* Chicken, turkey or rabbit.
*d* Pork, beef, lamb, veal, mutton or goat.
*e* Sugar, candies, pastries, fruit juices and soft drinks.
**PBDA**: Plant-based dairy alternatives.
**PBMA**: Plant-based meat alternatives.
