# Supplementary material for: Environmental and economic impact of a vegan versus traditional mediterranean diet: OMNIVEG study
Source: Eur J Nutr. 2026 Mar 17;65(3):97. doi: 10.1007/s00394-026-03939-3 (PMC12995930; doi:10.1007/s00394-026-03939-3)
Supplement: Supplementary file 3 — Supplementary Material 3 [file 394_2026_3939_MOESM3_ESM.docx]

| **End-point** | **Human health (DALY)** | | | **Ecosystems (species.yr)** | | | **Resources (USD2013)** | | **Food cost (€)** | |
| --- | --- | --- | --- | --- | --- | --- | --- | --- | --- | --- |
|  | *MedDiet* | *Vegan MedDiet* | | *MedDiet* | *Vegan MedDiet* | | *MedDiet* | *Vegan MedDiet* | *MedDiet* | *Vegan MedDiet* |
| Cereals | 1.49 x 10^-6^ | | 1.95 x 10^-6^ | 7.14 x 10^-9^ | | 8.23 x 10^-9^ | 2.46 x 10^-2^ | 2.97 x 10^-2^ | 0.74 | 0.80 |
| Vegetables | 2.09 x 10^-6^ | | 1.36 x 10^-6^ | 6.65 x 10^-9^ | | 4.73 x 10^-9^ | 9.63 x 10^-2^ | 6.44 x 10^-2^ | 1.06 | 1.36 |
| Fruits | 1.15 x 10^-6^ | | 1.08 x 10^-6^ | 4.32 x 10^-9^ | | 4.45 x 10^-9^ | 3.66 x 10^-2^ | 3.38 x 10^-2^ | 0.95 | 1.45 |
| Dairy | 1.50 x 10^-6^ | | 0.00^***^ | 1.04 x 10^-9^ | | 0.00^***^ | 3.33 x 10^-2^ | 0.00^***^ | 1.01 | 0.00^***^ |
| PBDA | 3.96 x 10^-8^ | | 5.04 x 10^-7***^ | 2.93 x 10^-10^ | | 3.24 x 10^-9***^ | 1.65 x 10^-3^ | 2.58 x 10^-2***^ | 0.03 | 0.85^***^ |
| Meat | 4.69 x 10^-6^ | | 0.00^**^ | 3.13 x 10^-8^ | | 0.00^***^ | 7.16 x 10^-2^ | 0.00^***^ | 2.18 | 0.00^***^ |
| PBMA | 0.00 | | 2.30 x 10^-7*^ | 0.00 | | 1.67 x 10^-9*^ | 0.00 | 9.14 x 10^-3*^ | 0.00 | 0.74^***^ |
| Eggs | 8.64 x 10^-7^ | | 0.00^*^ | 5.71 x 10^-9^ | | 0.00^*^ | 1.33 x 10^-2^ | 0.00^*^ | 0.32 | 0.00^***^ |
| Fish | 2.78 x 10^-6^ | | 0.00^*^ | 8.93 x 10^-9^ | | 0.00^*^ | 8.40 x 10^-2^ | 0.00^*^ | 1.02 | 0.00^***^ |
| Legumes | 1.78 x 10^-7^ | | 7.95 x 10^-7**^ | 2.04 x 10^-9^ | | 8.34 x 10^-9**^ | 6.63 x 10^-3^ | 2.98 x 10^-2**^ | 0.27 | 0.92^***^ |
| Nuts | 5.08 x 10^-7^ | | 7.36 x 10^-7^ | 5.01 x 10^-9^ | | 7.13 x 10^-9^ | 1.26 x 10^-2^ | 1.85 x 10^-2^ | 0.53 | 0.74^**^ |
| Fats | 3.12 x 10^-7^ | | 3.45 x 10^-7^ | 3.31 x 10^-9^ | | 3.43 x 10^-9^ | 7.63 x 10^-3^ | 8.18 x 10^-3^ | 0.21 | 0.21 |
| Others | 2.88 x 10^-7^ | | 2.23 x 10^-7^ | 1.39 x 10^-9^ | | 1.17 x 10^-9^ | 7.75 x 10^-3^ | 4.40 x 10^-3^ | 0.39 | 0.18 |
| Supplementation | - | | - | - | | - | - | - | - | 0.02* |

**Table S2**. End-point environmental and economic impacts of food groups according to dietary intervention.

The data are shown as mean daily values. DALY, Disability Adjusted Life Years; MedDiet, Mediterranean diet; PBDA, plant-based dairy alternatives; PBMA, plant-based meat alternatives; Vegan MedDiet, Vegan Mediterranean diet. Environmental impacts were assessed via Life Cycle Assessment using ReCiPe 2016 v1.1 characterization method. Comparison between groups by paired Student´s t-test.  ^*^ p< 0.05, ^**^ p<0.01, ^***^ p<0.001.
